# Global challenges of implementing human papillomavirus vaccines

**DOI:** 10.1186/1475-9276-10-27

**Published:** 2011-06-30

**Authors:** Janice E Graham, Amrita Mishra

**Affiliations:** 1 Department of Bioethics, Dalhousie University, Faculty of Medicine, 1459 Oxford Street, Halifax, Nova Scotia, B3H 4R2, Canada; 2 Technoscience and Regulation Research Unit, 1459 Oxford Street, Halifax, Nova Scotia, B3H 4R2, Canada

**Keywords:** Human Papillomavirus, cervical cancer, vaccine, immunization, equity, gender

## Abstract

Human Papillomavirus vaccines are widely hailed as a sweeping pharmaceutical innovation for the universal benefit of all women. The implementation of the vaccines, however, is far from universal or equitable. Socio-economically marginalized women in emerging and developing, and many advanced economies alike, suffer a disproportionately large burden of cervical cancer. Despite the marketing of Human Papillomavirus vaccines as *the *solution to cervical cancer, the market authorization (licensing) of the vaccines has not translated into universal equitable access. Vaccine implementation for vulnerable girls and women faces multiple barriers that include high vaccine costs, inadequate delivery infrastructure, and lack of community engagement to generate awareness about cervical cancer and early screening tools. For Human Papillomavirus vaccines to work as a public health solution, the quality-assured delivery of cheaper vaccines must be integrated with strengthened capacity for community-based health education and screening.

## Introduction

In 2006, Gardasil^® ^or Silgard^® ^(Merck), an adjuvanted vaccine against four Human Papillomavirus (HPV) types, was licensed for market in the Gabon, followed by the US, Canada and more than 100 other countries [1].(See additional file 1: appendix) Initial recommendations for use of Gardasil^® ^in Canada and the US included pre-coitarchal 9-13 year-old females and 14-26 year-old females even if already sexually active, with a history of cervical abnormalities or prior HPV exposure [2,3]. The vaccine was not recommended for females who were pregnant or less than 9 years of age. For women older than 26 years, immunization could be considered according to individual circumstances. In May 2007, another HPV vaccine, Cervarix^™ ^(GlaxoSmithKline) became available in Australia for females aged 10 to 45, in September 2007 in the European Union (EU) and in the US in 2009 for females aged 10 to 25 [4-6]. In 2009, the U.S. Food and Drugs Administration (FDA) licensed the use of Gardasil^® ^for 9-26 year-old males against genital warts caused by HPV 6 and 11 [7].

Gardasil^® ^and Cervarix^™ ^are prophylactic vaccines for the primary prevention of HPV types 16 and 18 (implicated in 70 per cent of cervical cancers). Additionally, Gardasil^® ^protects against HPV types 6 and 11 (implicated in genital warts, which are non-lethal but painful and hard to treat) [8-10]. A 3-dose intramuscular administration (currently costing approximately US$ 360) at 0, 1-2 and 6 months is required [6]. The vaccines generate a relatively robust immune response against targeted HPV types in 15-25 year old females who are pre-coitarchal and/or DNA and serologically negative for the targeted HPV types [11,12]. For both vaccines, the immune response in older women (25-45 years) is stronger than natural infection levels but less than that in 9-15 year olds [13,14].

HPV vaccines have been formally recommended, although not uniformly adopted, for large-scale use in the public sector healthcare systems and national immunization programmes of the wealthier countries of Europe, North America and Australasia [1,15,16]. In developing countries, however, HPV vaccines are not available through national immunization programs. (Following the usage of the International Monetary Fund, and in the absence of other established naming conventions, we apply the term "developing country" to emerging and developing economies [17].) Cost is a leading barrier to equitable delivery of HPV immunization in developing countries, where limited health budgets must address multiple contending priorities [18]. The high vaccine cost can be linked to the monopoly pricing power of vaccine manufacturers seeking to recover high development costs. Their retention of exclusive patent rights and their power to keep vaccine prices high are aided by the absence of compulsory licenses, which could authorize the competitive development of cheaper biogenerics through developing country manufacturers [19-21]. Public sector funding, the aid of vaccine funding consortia such as the Global Alliance for Vaccines Initiative (GAVI), and suitable technology transfer mechanisms are crucial to make HPV vaccines available at affordable prices in developing countries [19-21].

### Analytical approach

Taking a science studies approach, we regard vaccines as socio-technical objects that have technical, cultural, historical, economic, geo-political, and ethical dimensions [22]. While vaccination is a technical means to achieving immune protection against disease, immunization encompasses technical means and socio-political settings, actors, plans, objectives and results. Thus, through this paper, we adopt the term 'immunization' in preference to 'vaccination.'

Furthermore, we draw on the Erickson-DeWals-Farand analytical framework to examine the implementation of HPV vaccines [23]. That framework suggests that the inclusion of a vaccine in a publicly funded immunization program requires consideration of both technical and social factors, and of their interrelationship. These factors include disease burden, vaccine efficacy, safety, and immunogenicity, absolute cost and marginal cost-effectiveness ratios, immunization strategy, implementation feasibility, delivery resources, and dose schedules, and equally important, public knowledge and acceptance, and the political implications of immunization strategy, such as mandatory or elective immunization [23]. HPV immunization exemplifies vaccine politics. It not only activates debate on gender, freedom, safety, responsibility and management of adolescent sexuality, but also raises questions surrounding equity in delivering new vaccines to the world's poorest regions.

While the Erickson-DeWals-Farand framework was developed for the Canadian context, we extend its scope to examine the challenges for HPV vaccine implementation, particularly in developing countries. Vaccines are transnational commodities, subject to national and international regulatory decision-making related to standards of safety, efficacy and quality. As with all health technologies, however, vaccines are not created or distributed equally. Diseases endemic in developing countries remain under-researched. Even when effective preventions and therapies are discovered, they are often unavailable to people in developing economies, including those in high-income countries. HPV immunization is a case in point. Currently, developing countries bear about 80 per cent of the global mortality from cervical cancer, i.e., an estimated 242,000 compared to 33,000 deaths in high-income countries [24-26]. Given the gaps in secondary prevention, HPV immunization would seem the obvious intervention to control mortality from cervical cancer in developing countries. Unfortunately, the implementation of HPV vaccines in resource-poor regions faces not only the barriers of vaccine cost, competing health priorities, and public acceptance, but also the systemic lack of infrastructure that inhibits secondary prevention through cervical screening. Further, lack of awareness and social restrictions on gynecological examination prevent women from accessing screening services when available.

### The challenges of secondary prevention

In this section, we elaborate on the infrastructure and social barriers to secondary prevention of HPV-related disease. These gaps are persistent and will continue to hamper immunization strategies, even when HPV vaccines are made readily available in developing countries.

Of the over-100 classified genotypes of HPV, over 40 types can infect the upper respiratory-digestive tracts and anogenital areas. In most cases, HPV infection is cleared or becomes undetectable, causing no disease; however, persistent HPV infection is causally related to the development of cancers and genital warts. Over 99% of cancers of the uterine cervix are attributed to persistent infection by HPV 16, 18, 31, 33, 35, 39, 45, 51, 52, 56, 58, 59, 66 and 68, in that order of frequency. HPV is also associated with external genital warts, respiratory papillomatosis, and head, neck, anal, penile, vulvar and vaginal cancers [27-31].

Cervical cancer is generally marked by a long latency period, during which lesions progress through identifiable stages to invasive growth. Secondary prevention involves screening of asymptomatic women for detection, triage, management and monitoring of precancerous abnormal cells and atypical squamous cells of undisclosed significance (ASCUS). Screening methods include conventional cytology (Papanicolaou or 'Pap' smears, microscope examination of slide-mounted cervical cell samples), Liquid-Based Cytology (LBC), Visual Inspection with Acetic Acid (VIA), Visual Inspection with Lugol's Iodine (VILI), and more recently HPV DNA tests for triage of women with ambiguous cytology to colposcopy or for surveillance of women older than 30 years for the presence of high-risk HPV DNA [32,33].

Although there is still no treatment for HPV, since the 1950s, cytologic screening has been a key tool in prevention of cervical cancer. In high-income economies, organized programs for screening and post-screening treatment have reduced morbidity and mortality from cervical cancer through standardised systems of screening, monitoring, and recall-and-reminder [24-26,32-34]. Resource-poor settings in developing countries, however, often lack primary physicians, screening equipment (e.g., colposcopes), trained laboratory personnel (e.g., cytotechnicians), and necessary expertise and capacity for screening quality assurance [34]. The lack of effective screening programs is often combined with inadequate knowledge about cervical cancer (causes, symptoms, progression and treatment), even among healthcare workers [35]. In emerging economies (e.g. Malaysia) and high-income Asian economic regions (e.g. Hong Kong), cervical screening can still be hampered by embarrassment surrounding sexually transmitted infections (STI) and gynecological examination, as well as inadequate awareness about HPV-related risks and protections [36,37].

Even in high-income countries, availability of screening services does not always translate into accessibility for socioeconomically marginalized and vulnerable girls and women. In Canada, the US, the UK, and Australia cervical cancer is higher among indigenous and minority women, who experience the health effects of poverty, inadequate insurance, linguistic barriers, insufficient knowledge of health needs and risks, lack of trust in health services, and shame regarding gynecological consultations (conversation) and examination [38-45].

The labor-intensive assessment of Pap smears is associated with false negatives and low sensitivity, which require women to repeat the test. The American Congress of Obstetricians and Gynecologists (ACOG) recommend that screening start at age 21, to be repeated once every 2 years until age 30 and then every 3 years until 65-70 [46]. Despite repeated efforts to standardize cytologic reporting guidelines and screening practice, there are knowledge gaps and disagreements over the management of patients, in particular those with ASCUS and low-grade lesions, care for women older than 65 years, and the effects of HPV immunization on screening initiation and intervals [47-51].

Screening repeats demand follow-up compliance, which is a particular challenge in remote impoverished rural areas, where women must schedule time away from necessary daily tasks (such as herding, fetching water or firewood) or travel long distances to clinics to be tested. Ensuring compliance requires maintaining screening registries and establishing mechanisms for reminders and follow-ups that do not provoke anxiety or cause embarrassment among women [52]. Inadequate networks, personnel and expertise inhibit public education, disease surveillance and follow-up monitoring in resource-poor settings.

The implementation of the other screening methods also faces challenges. Although HPV testing is less affected by subjectivity or artifact than cytology, it requires more laboratory facilities, costs more than Pap tests, and can be difficult to accomplish in developing countries. Further, HPV testing has higher sensitivity but lower specificity than cytology, implying fewer false negatives but increased false positives and over-triaging to colposcopy. This can increase resource demands on healthcare and anxieties and physical burdens for patients. Although new and cheaper HPV tests are being developed, they are not yet available for widespread use in developing countries [53-55]. Although LBC allows a combination of cytology and HPV testing, cytology itself remains error-prone. Inexpensive visual screening procedures such as VIA and VILI are easier to implement in developing settings and local health workers can be trained to do them. The procedures, however, lack guidelines and the readings are highly subjective, error-prone and not easily reproducible [56].

Women in remote rural areas are at greater risk of cervical malignancies because they have limited access to cervical screening and appropriate treatment. Further, they frequently present for the first examination with late stage malignancies and co-morbidities such as malnourishment, anemia, malaria and HIV/AIDS [57-59]. In developing countries, the resource limitations of medical services, including oncology, constrain 'structure' (the capacity of the medical system to manage disease). Structural limitations constrain 'access' (availability, spatial accessibility, affordability, accommodation, acceptability, and awareness) and negatively impact 'process' (the quality of care from entry to discharge, and post-discharge) and 'outcomes' (the effects of medical intervention on survival, quality of life, patient awareness and behavior) [60].

Late presentation and the lack of therapeutic options (e.g., cryotherapy or loop electrosurgical excision for cervical intraepithelial neoplastic lesions; cold knife conization and simple to radical hysterectomy for micro-invasive cancers; irradiation and/or chemotherapy for more advanced stages) probably explain the observed 5-year survival rates of less than 25% in Uganda and Zimbabwe. This is in contrast to 60-75% survival rates in high-income countries. Women may be ashamed of or resigned to their condition. They may not obtain or complete available treatment because of stigma associated with hysterectomy or the fear of hair loss, for example, from chemotherapy [60,61].

### Vaccine rhetoric: Unpacking the claims

Although HPV immunization has the potential to address the challenges of screening, a number of social and policy barriers still prevent its implementation as a public health solution in developing economies. Public awareness, for example, would facilitate both primary and secondary prevention by tackling issues such as embarrassment surrounding STIs and the public reception of HPV immunization. Vaccines are most often given to healthy, young (immunologically naïve) individuals. The administration of a vaccine is invasive, can be painful, and has the potential of adverse events; harms and benefits must be weighed. Efforts to communicate and exchange information could promote acceptance and uptake. Commercial rhetoric, however, has taken precedence over public health education about HPV, its related diseases, and the prophylactic effects and limitations of HPV vaccines.

Rhetoric has played a major role in industry marketing and how the entire range of stakeholders, including industry, scientists, health care providers and the public, view the vaccine and HPV implementation. The HPV vaccine has become a contested object where industry advertising and profit motives have pre-empted its public health value.

Mandatory HPV immunization has been criticised as a violation of parental autonomy for the prevention of a non-casually transmitted infection, a misuse of taxpayers' money for a vaccine of unknown effectiveness at the expense of more pressing public health issues, an unnecessary addition to overloaded vaccine schedules, a promotion of teenage promiscuity, and a devaluation of abstinence messages. Mandatory immunization supporters argue that abstinence is ineffective in protecting against STI, that secondary prevention of cervical cancer has irremediable gaps, that there are already mandates for Hepatitis B Virus (HBV) immunization, and socio-economically vulnerable women must be protected against cervical cancer [62-64].

HPV vaccines are marketed as a women's right, choice and duty for health security, exemplified in the Gardasil 'I choose' commercials [65,66]. Socioeconomic realities pose barriers to access and choice, however, for immunization and screening [67]. Additionally, HPV and cervical cancer affect the families and social and economic networks of women who succumb to the disease because they lack access to both prevention and treatment. The advertising around HPV vaccines conflates HPV infection, which is widely prevalent and easily transmitted, with cervical cancer, which is "a relatively *rare consequence of *a *common infection*" [68: 365]. On occasion, the scientific literature tends to equate HPV immunization with the 'end of cervical cancer' [69,70].

Pharmaceutical advertising of the so-called 'cervical cancer vaccine' resonates with an operative dilemma for public health 'social marketing': Is the acceptability of the vaccine improved by presenting it as 'anti-cancer', instead of 'anti-STI' [71]? On the one hand, Gardasil receives high acceptance from parents who wish to protect the health of their children. On the other hand, parents worry that their children may interpret their acceptance of the vaccine as approval of sexual activity. Parents are concerned that their children may see HPV immunization as blanket protection against the effects of sexual risk-taking. Parents who reject the vaccine may believe that their children's 'safe' or 'moral' behavior is adequate protection against STI. In the absence of balanced public health information, media reportage has stoked community concerns about the effects of HPV vaccines on teen behavior [9,72-76]. In the public health perspective, health is a public good rather than a means for profit. The public funding, delivery and allocation of a vaccine are informed by the idea of immunization as a public good. The public profile of the HPV vaccine has been undermined by infomercials that create the impression of a personalized 'drug against risk,' an object for commercial profit and consumer desire, separate from public health considerations of community, equity, and accessibility [65,77].

The high cost of the vaccine has compelled public payers to ration its subsidization to unexposed adolescent females with the expectation that high uptake will build herd immunity. This has resulted in HPV, HPV-related disease, and HPV vaccine being marked as 'women-only,' despite the fact that men also get and transmit the virus. The prescription of 'HPV immunization for girls' to ensure 'herd immunity' suggests that HPV is an exclusively heterosexual concern. HPV also affects lesbian, gay, bisexual and transgender (LGBT) people. HPV vaccine commercials and public health messages focus on cervical cancer, to the exclusion of throat, anal and penile cancer [78]. Despite the reported prevalence of oncogenic HPV type 16 in anal HPV infection, anal Pap smears are hindered by silence, homophobia, lack of awareness, fear of stigma, and unaddressed technological gaps [78]. Even in many high-income countries, LGBT people may not seek sexual health advice, or screening services due to fear of outing and its consequences [79].

Focus on school-based immunization of preadolescent girls removes attention from the risk faced by older women from invasive disease due to persistent HPV infection [30]. In Canada and the US, women above 26 years of age are not vaccine eligible [2,3]. Women in lower socio-economic brackets would not be able to pay for the vaccine at its current cost. Funding for cervical screening programs for older, socioeconomically vulnerable women should be strengthened. Exclusive strategic focus on HPV immunization for younger women should not weaken attention to the health needs of older women.

It will take at least 20 years of targeted immunization of adolescent cohorts before there is a measurable reduction in cervical cancer incidence [14]. In the short term, HPV immunization may affect screening for precursors to invasive disease [14,80,81]. Cytologists may need re-training to accommodate new sensitivity and specificity from higher rates of negative slides per number viewed. HPV tests, if available at lower prices, may prevent harms from reduced cytologic accuracy. The creation and integration of immunization and cancer registries would facilitate monitoring of disease and vaccine effectiveness, identification of vulnerable populations (mapping vaccine uptake and high-risk behaviour), and the possible revision of screening guidelines [82-84]. Even in well-resourced settings, however, privacy laws and issues related with transferring records from paper to electronic formats may hamper surveillance [85]. These problems are exacerbated in developing countries without the infrastructure or capacity for adequate population health monitoring and databases.

### Comparing Hepatitis B and human papillomavirus immunization

The clinical efficacy of both HBV and HPV vaccines is in their ability to block the acquisition of viruses that are transmitted through non-casual intimate physical contact (with HBV additionally blood-borne) [28,68,86]. Real-world effectiveness, however, is in the prevention of acute and chronic conditions and cancers induced by persistent viral infection, e.g., fulminating hepatic necrosis, chronic infection with liver failure and primary hepatocellular carcinoma from HBV and papillomatosis and oropharyngeal and anogenital carcinomas from HPV [27-31,68,86]. The clinical effectiveness of a vaccine dovetails with cost-effectiveness when it is delivered to populations with the greatest burden of disease. In this area, HBV immunization has fared better than HPV immunization and illustrates the important role of multilateral partnerships in making new vaccines available to vulnerable populations in developing countries.

At the time of introduction, both vaccines have raised concerns about cost, cost-effectiveness, public acceptability, and priority for public health [86,87]. A combination of factors made the initially expensive HBV vaccine (around US$ 100 during the early 1980s-1990s) available for use in childhood immunization programs in developing countries [86]. These factors included technology transfer for manufacturing a cheaper biogeneric at US$ 1.00 per dose, feasibility demonstration projects of the International Task Force on Hepatitis B Immunization, and the intervention of GAVI to make the vaccine available in eligible countries [86]. In 1994, the World Health Organization (WHO) incorporated HBV vaccine into the Expanded Program on Immunization (EPI).

GAVI may be able to subsidize HPV vaccines for delivery in the poorest countries, provided vaccine manufacturers sell the vaccine to GAVI at lower prices [88]. This process involves several stages: first, the vaccine price reduction for country purchase; second, GAVI Board approval for vaccine funding, conditional on availability of funds; and finally, decision-making by GAVI-eligible countries on the inclusion of HPV vaccine in national immunization programmes [88]. Without cost reduction, HPV vaccine cannot be offered for 9-13 year-olds through the WHO EPI program, which provides the infrastructure and public trust to have some success [35]. Such programs are not available in many emerging economies. Thus, although some individuals may be able to purchase the vaccine, it remains out of reach to most vulnerable women.

In the US, HBV vaccine mandates have been passed, delivering an otherwise expensive vaccine to children prior to their school entry. A comparison of the differing histories of HBV and HPV vaccines in the US illustrates the importance of institutional credibility, dialogue, and regulatory decisions related to licensing and implementation.

It took seven years for the HPV vaccine to go from FDA approval in 1986, to the US Advisory Committee on Immunisation Practices (ACIP) recommendations in 1991, to mandated requirement for day care attendance, in 1993. This time, along with its cost-reducing inclusion in the Vaccines for Children (VFC) program in 1994, provided the opportunity for the public to become aware of HBV immunization and accept its mandate [89]. In contrast, the rush to mandate Gardasil in 2007 (Texas, subsequently overturned) after its 2006 licensing, accompanied by an aggressive marketing campaign, attracted criticism and controversy. The intrusion of pharmaceutical interests into health policy was suggested [64,89-91]. This speed contributed to suspicions rather than building public awareness and acceptance of HPV immunization. After much legislative debate in several US states, only Virginia and the District of Columbia passed HPV mandates, with extensive opt-out provisions [89,91].

Immunization against HPV, unlike that against HBV, specifically and conspicuously targets female adolescents [92]. Socially entrenched anxieties about female sexuality are activated to a greater extent and influence the public reception of HPV immunization [62,63]. These views should be debated through a free and, if necessary, even protracted, exchange of views and information. Accelerated vaccine rollouts, regardless of intention, limit the possibility of debate and exchange of views.

### From laboratories to people: Complex itineraries for HPV vaccines

The successful development and market authorization of vaccines do not in themselves realize the public health purposes of immunization. A vaccine, no matter how innovative and efficacious, serves public health only if it is a safe, affordable, accessible and cost-effective intervention. There are multiple barriers to making HPV vaccines available for use in medically-underserved developing, economies. In general, the delivery of vaccines in developing countries faces a wide range of socio-technical challenges that include lack of institutional capacity for monitoring of pre-market vaccine quality and safety and post-market surveillance for adverse events following immunization (AEFI), cold chain difficulties in vaccine delivery to populations in remote rural areas, risk of infection during needle-administration, and fear of needles [23]. With HPV vaccines, there are other, specific challenges.

As discussed, the availability of HPV vaccines in developing countries is particularly hampered by high vaccine costs, bottlenecks of technology transfer for developing cheaper subsequent entry vaccines, low acceptability of vaccines against STI for pre-adolescents, and the problems of integrating HPV vaccines into the formularies of country EPIs [19-21]. These challenges are summarized in Table 1. For policy makers in developing countries, the prevention of a disease that manifests years in the future has less political urgency than the immediate prevention of conspicuous diseases such as poliomyelitis, tuberculosis, or HIV/AIDS. For an individual, hunger and inadequate shelter trump concerns about a silent, slow-developing cancer. The risk-benefit profiles that drive health decision-making in a well-off economy do not apply in contexts where political violence, forced migration, epidemics (e.g., cholera, meningitis), drought, floods, crop failure, and starvation threaten daily survival. Despite promising clinical data, questions persist about immunogenic duration and the need for boosters on HPV vaccine effectiveness [93]. These issues have significant cost-effectiveness implications for poorer individuals, communities, and countries. The currently available vaccines target 16 and 18, two of the 15 most prevalent high-risk HPV types. Among the types not targeted, HPV types 45 and 31 are prevalent, respectively, in sub-Saharan Africa and Central/South America. The development of effective multi-antigen HPV vaccines, however, may be challenged by cross-reactivity between included antigens [27]. The early age of immunization (9 years for Gardasil^®^) also remains contentious. Despite clinical data that supports targeting unexposed recipients, parental beliefs about the appropriateness of pre-adolescent immunization limit the uptake of HPV vaccines among pre-teens. Vaccine decisions are made not only by designated policy-makers but also by the public, whose knowledge, attitudes and beliefs (KAB) are key factors in vaccine acceptance. Consistently, across a wide range of countries and ethnicities, KAB studies report socio-cultural barriers to HPV vaccines. These include lack of awareness about HPV effects and protections, parental and physician unwillingness to vaccinate or to discuss sex and STI with preadolescent females, concerns about vaccine costs and uncertain duration of effectiveness, fear of unethical motives and practices in vaccine trials, fear of needle pain, adverse effects (e.g., infertility and physical disfigurement), socially unacceptable vaccine ingredients (e.g., alcohol, a concern for Muslims), stigma and sexual disinhibition [94-110].

**Table 1:** From Laboratories to People: Barriers in HPV vaccine implementation

INNOVATION	PROCUREMENT AND DELIVERY	POLICY FORMULATION	SOCIAL CHALLENGES (Knowledge, attitudes, beliefs)
Patent monopolies [19].	Inadequate regulatory mechanisms, resources and infrastructure, i.e., for reporting of adverse events and post-market surveillance [23,129].	High vaccine costs [18].	*Parents and adolescents *[94-97,99-109]
Scarcity of instruments and models for technology transfer of inexpensive biogenerics [19,21].	Cold chain issues, preventing quality- assured and controlled transportation and storage of vaccines [23,129].	Competing health priorities (e.g., HIV/AIDS, malaria) [105].	Perceived low HPV/STI susceptibility.
Gaps in multilateral funding for vaccine procurement [20].		Controversies over HPV vaccine mandates [64-66,86,89-91].	Unwillingness to discuss sex and STI.
		Inadequate knowledge exchange about STI risks and need for prevention [110,113,114].	Perceived inappropriateness for pre-adolescents.
			Stigma, loss of privacy.
			Concerns about adolescent promiscuity and beliefs in moral education and marital monogamy.
			Suspicion of commercial motives and unethical vaccine trials.
			Worry about vaccine ingredients and adverse effects.
			Needle fears.
			Gaps in availability and access. Competing life priorities and pressures.
			Vaccine costs and duration of effectiveness.
			*Healthcare providers *[98,110]
			Unwillingness to endorse vaccine, discuss sex and STI, or to stock vaccine
			Lack of personnel and facilities for vaccination and heavy case loads in clinics.

Uncertainties and fears associated with unknown substances in the vaccine can discourage some. Rumors of adverse (or rumoured deliberate) effects, such as sterilization, led to the failure of polio vaccine initiatives in northern Nigeria [111]. A 3-dose HPV vaccine schedule poses compliance burdens. Information written in a vaccine booklet is not accessible to women without literacy. Further, keeping a vaccine booklet intact and legible may be a problem for those living in huts with termites and leaking roofs [112].

Even if inexpensive HPV vaccines could be developed, manufactured, subsidized and supplied with quality assurance, parents may not always accept immunization of their pre-adolescent children for an STI [71]. Challenges of knowledge translation that have hampered efforts to promote cervical screening may carry over to HPV immunization. For instance, in a Hong Kong study of attitudes to HPV immunization, Chinese participants thought that HPV caused disease of the nipple, because the Chinese characters used for HPV translated as 'breast' or 'nipple' [95]. Misunderstandings such as these can hamper both the perceived need for health protections and efforts to obtain them. Physicians, who play a key role in knowledge exchange and uptake of health protections, report confusion about HPV, HBV, Herpes Simplex virus (HSV), and Human Immunodeficiency Virus (HIV) [113,114]. KAB studies demonstrate the absence of a reliable body of publicly accessible information on cervical cancer and its link to HPV. Individuals may not perceive themselves at risk for HPV-related disease, especially when they are not having sex, or are in monogamous marital relationships [94,95,99,106,108]. The lack of awareness of HPV transmission and health risks may be exacerbated by the length of time from infection to disease. In strongly male-dominated societies, values of shame and honour prevent women's matters, including female anatomy and STIs, from being openly discussed and define 'modest womanhood' [115]. These are powerful social barriers to effective dialogue about HPV and cancer. Indian physicians, for example, are reluctant to discuss STIs and prophylaxis with female patients or their families and see HPV immunization as an unnecessary addition to already heavy caseloads [110].

The integration of screening and immunization into public health education is critical to efforts to control cervical cancer [116]. The evidence generated by KAB studies needs to be translated into community-based programs about the risks of HPV and the need to take action against it. Text-heavy or hard-to-access web-based information will not reach the illiterate or those without computers [113]. Simple messages through low-cost outreach strategies (e.g., street theater, door-to-door visits by community workers, discussions at markets and bus stops) could more effectively engage the community [113,117]. Messaging must convey that both men and women are sufferers and carriers of HPV and that HPV is neither an exclusively female problem nor a matter of shame for women. Information about HPV and cervical cancer need to target males, who in traditional societies are often key decision makers, even in deciding women's visits to clinics [95,103,107,108,112].

### HPV immunization: Comparing implementation scenarios

Internationally, the implementation of HPV immunization has had a variety of outcomes, associated with a constellation of factors affecting health practicalities, policies and priorities.

Australia, Canada and the UK have publicly funded programs that provide HPV immunization for peri-adolescent girls through school based and catch-up programs [80,118,119]. In the EU countries offering subsidized HPV immunization, funding modalities and target populations are heterogeneous [120,121]. Vaccine delivery in the EU is school-based, or on-demand from general practitioners or public health clinics, with or without a letter of invitation [120,121]. While the vaccine is offered through public health systems in Denmark, France, Germany, Italy, Spain, the Netherlands, and the UK, the Austrian government decided against including the vaccine in publicly funded immunization programs. They reasoned that it would not be as cost-effective as Pap testing; even with a high and sustained uptake among both boys and girls, there would be no appreciable reductions in cervical cancer for many decades [121]. In the UK, reduced acceptability of the HPV vaccine may have been affected by the now-debunked link between autism and Measles-Mumps-Rubella (MMR) vaccine [121]. Few Eastern European countries have adopted HPV into their national immunization programs. These countries also lack organized screening programs, possibly indicating their lack of resources [16].

In the US, HPV immunization is available outside the school system. Uninsured adolescents in the age-eligible cohort can obtain the vaccine free of cost through the VFC program [89]. For others, HPV immunization depends either on individual ability to buy the vaccine or on the extent of insurance coverage. There are no established guidelines or standards for adolescents' visits to doctors. Adolescents are confronted by waning immunity as well as poorer compliance with clinic visits required by multi-dose schedules [117,122,123]. In Canada, HPV immunization is delivered as part of the standard school vaccine formulary through public-health mechanisms to girls in Grades 7 and 8 [124]. Immunization requires informed and voluntary parental consent [125]. According to Canada's National Advisory Committee on Immunization (NACI), a rationale for targeting Grade 7 girls for HPV immunization is that school-dropouts and sexual activity are still relatively limited in that cohort [2]. In Canada, HPV immunization is delivered alongside accepted and normalized vaccines (e.g., HBV) through established public-health systems [92,124,125]. This integration of HPV vaccine into existing delivery systems may have softened the controversy and operational problems of a federally-directed vaccine roll-out, whose speed precluded prior consultation and knowledge-sharing with Canadian stakeholders at the provincial and territorial levels [77,126,127].

In developing countries, HPV vaccines are not offered as part of national immunization schedules. Immunization costs are not covered and vaccine uptake may be determined by the market (and marketing). Although school-based delivery platforms have a good performance record and are relatively equitable, they may have less success in many developing countries. Even where schools are available, young girls are often unable to attend due to obligations both in and outside the home. These programs also bypass street-dwelling youth in developing and advanced economies [128]. Catch-up immunization programs are only marginally successful. Mandatory HPV immunization, although effective, would strain the health budgets of developing countries.

## Conclusion

Although HPV vaccines represent a promising biological innovation, they will yield the greatest public health benefits only when introduced at affordable prices into a context of robust healthcare infrastructure. Determining which vaccines to include in a public health program requires capacity-intensive infrastructure for the regulation, production, and post-market surveillance of vaccines [129]. In the event that HPV vaccines become available at lower prices in developing countries, gaps in capacity in these countries lends difficulties to effective decision-making, delivery, and surveillance.

HPV vaccine remains unavailable to poor and vulnerable women. Those most able to access HPV immunization may be also those more able to access cervical screening [80]. The result is a widening of health disparities, in both developing and developed countries. Socioeconomic inequalities persist in the inability of vulnerable women to access health services, even in countries with a national health service. As competition reduces costs and HPV vaccines become accessible in poorer regions, it will remain important for individuals, communities and health care providers to engage in dialogue about HPV transmission, risks, disease, and prevention through cervical screening as well as immunization.

## List of Abbreviations used

ACIP: Advisory Committee on Immunization Practices; ACOG: American Congress of Obstetricians and Gynecologists; AEFI: Adverse Events Following Immunization; ASCUS: Atypical Squamous Cells of Undisclosed Significance; EPI: Expanded Program in Immunization; EU: European Union; FDA: Food and Drugs Administration; GAVI: Global Alliance for Vaccines Initiative; HBV: Hepatitis B Virus; HIV: Human Immunodeficiency Virus; HPV: Human Papillomavirus; HSV: Herpes Simplex Virus; KAB: Knowledge, Attitudes and Behavior; LBC: Liquid-Based Cytology; LGBT: Lesbian, Gay, Bisexual and Transgender; STI: Sexually transmitted infections; VIA: Visual Inspection with Acetic Acid; VILI: Visual Inspection with Lugol's Iodine; WHO: World Health Organization.

## Competing interests

The authors declare that they have no competing interests.

## Authors' contributions

AM and JG conceptualized and designed the study. AM selected the literature and drafted the manuscript. JG revised the manuscript. AM and JG read and approved the final manuscript.

## Authors' information

JG is Professor and Canada Research Chair in Bioethics, and Professor of Pediatrics (Division of Infectious Diseases) in the Faculty of Medicine at Dalhousie University. She is a medical anthropologist who draws upon epidemiology and technology assessment to study cultural, technical and moral issues related to health and the regulation of biotechnologies. She currently studies new and emerging vaccines, including vaccines against HPV, pandemic influenza, and the conjugate meningitis A vaccine for sub-Saharan Africa.

AM is a sociologist and a Postdoctoral Research Fellow at the Technoscience and Regulation Research Unit, Faculty of Medicine, Dalhousie University, Canada. Her current project 'The Administration of a Quadrivalent HPV Vaccine for Minors' is funded by the Canadian Institutes of Health Research (CIHR) and examines knowledge, attitudes and practices in HPV immunization in Nova Scotia, Canada.

## Supplementary Material

Additional file 1 **Appendix**. The data file is a word file containing an explanatory appendix with a statement regarding the use of a proprietary term. Click here for file
