# Supplementary material for: Global challenges of implementing human papillomavirus vaccines
Source: Int J Equity Health. 2011 Jun 30;10:27. doi: 10.1186/1475-9276-10-27 (PMC3143925; doi:10.1186/1475-9276-10-27)
Supplement: Additional file 1 — Appendix. The data file is a word file containing an explanatory appendix with a statement regarding the use of a proprietary term. [file 1475-9276-10-27-S1.PDF]

Appendix

This article includes three words (Gardasil®/Silgard® and Cervarix™) that are or are asserted to be proprietary terms or trademarks. Their inclusion does not imply they have acquired for legal purposes a non-proprietary or general significance, nor is any other judgment implied concerning their legal status.
